# A Textile Pile Debridement Material Consisting of Polyester Fibers for in Vitro Removal of Biofilm

**DOI:** 10.3390/polym12061360

**Published:** 2020-06-17

**Authors:** Yijun Fu, Qi An, Yue Cheng, Yumin Yang, Lu Wang, Haifeng Zhang, Yan Ge, Dawei Li, Yu Zhang

**Affiliations:** 1 College of Textile and Clothing, Nantong University, Nantong 226019, China; fuyj@ntu.edu.cn (Y.F.); anqi960110@163.com (Q.A.); cy970218@126.com (Y.C.); zhanghf@ntu.edu.cn (H.Z.); ntdxgeyan@126.com (Y.G.); 2 National & Local Joint Engineering Research Center of Technical Fiber Composites for Safety and Health, College of Textile and Clothing, Nantong University, Nantong 226019, China; 3 Key Laboratory of Neuroregeneration of Jiangsu and Ministry of Education and Co-innovation Center of Neuroregeneration, Nantong University, Nantong 226019, China; yangym@ntu.edu.cn; 4 Key Laboratory of Textile Science and Technology of Ministry of Education and College of Textiles, Donghua University, Shanghai 201620, China; wanglu@dhu.edu.cn

**Keywords:** textile pile debridement material, biofilm, wound care, plasma treatment, blood clotting

## Abstract

Biofilms formed on skin wound lead to inflammation and a delay of healing. In the present work, a novel textile pile debridement material was prepared and treated by plasma. Samples before and after plasma treatment were characterized by a series of methods, including scanning electron microscopy (SEM), atomic force microscopy (AFM), X-ray photoelectron spectroscopy (XPS), and water uptake capacity. Besides, mechanical, coagulation, and in vitro biofilm removal performances of the textile pile debridement material were evaluated, with a medical gauze as a control. The results demonstrate that the plasma treatment produced corrosions and oxygen-containing polar groups on the fiber surface, offering an enhanced water uptake capacity of the textile pile debridement material. In addition, compressive tests certify the mechanical performances of the textile pile debridement material in both dry and wet conditions. The results from a kinetic clotting time test suggest a favorable ability to promote blood coagulation. Furthermore, the results of an MTT cell viability assay, SEM, and confocal laser scanning microscopy (CLSM) illustrate that the textile pile debridement material demonstrates a more superior in vitro biofilm removal performance than medical gauze. All of these characterizations suggest that the textile pile debridement material can offer a feasible application for clinical wound debridement.

## 1. Introduction

Skin wound care has attracted worldwide attention because of its importance for public health and social development [1]. The first stage of wound healing is a local inflammatory response, which is a complicated pathophysiologic process and a result of the joint action of various biochemical mechanisms, which neutralize and remove harmful substances around the wound through physiological changes involving multiple factors [2]. Nevertheless, the duration of the inflammation phase is closely related to the gender, age, and health conditions of the patients themselves. Any negative factors, such as diabetes, hypertension, or anemia, may impede the inflammatory stage. In addition, the untimely and improper disposal of necrotic tissue and slough on the surface of the wound will cause infection and the abnormal formation of granulation tissue, resulting in a delayed wound healing [3]. Thus, the effective debridement of devitalized and contaminated tissue from the wound and the surrounding area is essential to reduce bacterial populations and prevent the infection of skin wounds [4,5].

Various kinds of wound debridement techniques are currently available in clinical practice, such as autolytic [6], enzymatic [7], biodebridement [8], mechanical [9], and surgical [10] debridement. A critical look at these options can explain their advantages but also limitations; autolysis is a simple and natural debridement approach, but it is time-consuming; enzymatic debridement, also known as chemical debridement, may cause a hypersensitive response in healthy tissue; biological debridement is highly selective, but has low acceptability for patients; mechanical debridement, as a nonselective method, always damages healthy tissue, causing pain; surgical debridement must be operated by professionals. Therefore, the exploration of safe, effective, and comfortable wound debridement materials is necessary to improve the work efficiency of medical staff and the living standard of patients.

Medical cotton, gauze, and bandages are the conventional textile-based materials applied for wound cleaning and debridement, due to their convenience in operation, as well as having an acceptable price for the public. However, patients frequently complain of pain when they are removed from or replaced on a skin wound [11,12]. A novel textile pile debridement material proposed in our previous study exhibits intrinsic superiorities over cotton gauze, owing to its inimitable advantages, such as a controllable structure design, soft texture, excellent flexibility, outstanding absorption capacity, and satisfactory biocompatibility, etc. [13]. In order to promote its clinical application, it is necessary to establish a fast evaluation system of the debridement function for textile pile debridement materials.

Biofilms, adhered to the surface of inert or active objects, are bacterial communities with constructive and coordinative functions [14]. Compared with planktonic bacteria, biofilms not only display a higher resistance to antibiotics, but also possess the ability to escape from the immune system of the host. The components of biofilms are complicated, including extracellular polysaccharides, proteins, lipids, DNA, etc. [15]. The formation of biofilms not only raises the production of pro-inflammatory cytokines, metalloproteinases, and neutrophils, but also aggravates the biological burden on wounds and breaks the normal physiological response process of wound healing. In addition, biofilms can release bacteria that migrate around and expand the area of bacterial colonization. Thus, this is considered as a vital factor causing chronic wound infection [16]. One of the purposes of debridement is to reduce bacterial colonization, control the level of inflammation, and suppress the spread of infection. Therefore, an in vitro biofilm removal test was proposed and set up to evaluate the debridement function of a textile pile debridement material.

In this work, a novel textile pile debridement material, consisting of polyester fibers, was designed and prepared. Afterwards, plasma treatment was implemented to further promote the liquid absorption ability of the textile pile debridement material. The compressive strength and elastic recovery of the textile pile debridement material in both dry and wet conditions were studied. Furthermore, the coagulation and in vitro biofilm removal performances of the textile pile material were assessed, with a medical gauze as a control, to explore its potential application for debridement.

## 2. Materials and Methods

### 2.1. Materials

Polyester staple fibers and draw textured yarn (DTY) were acquired from Jiangsu Chemical Fiber Co., Ltd., Wuxi, China. Biocompatible polyacrylate coating latex was supplied by Shanghai Huasen Chemical Co., Ltd.,Shanghai, China. Commercial medical gauzes from Winner Industries Co., Ltd., Shenzhen, China, were employed as control samples. *Staphylococcus aureus* (ATCC 25923) was provided by JK Microorganism Co., Ltd., China. Tryptic soy broth (TSB) was prepared with tryptone Lp0042, yeast extract Lp0041 (OXOID, Britain), and sodium chloride (Shanghai Lingfeng Chemical Reagent Co., Ltd., Shanghai, China). Methylthiazolyldiphenyl-tetrazolium bromide, 3-(4,5-dimethyl-2-thiazolyl)-2,5-diphenyl-2H-tetrazolium bromide (MTT) was purchased from Shanghai Bio-Gene Technology Co., Ltd., Shanghai, China. Fluorescein isothiocyanate (FITC) and propidium iodide (PI) were obtained from Sigma, USA. CaCl_2_, NaOH, glutaraldehyde, ethanol, and isopropanol were all purchased from Sinopharm Chemical Reagent Co., Ltd., Shanghai, China. All chemicals were analytical grade and were prepared with deionized water (DIW).

### 2.2. Fabrication and Plasma Treatment of the Textile Pile Debridement Material

**Figure 1:**
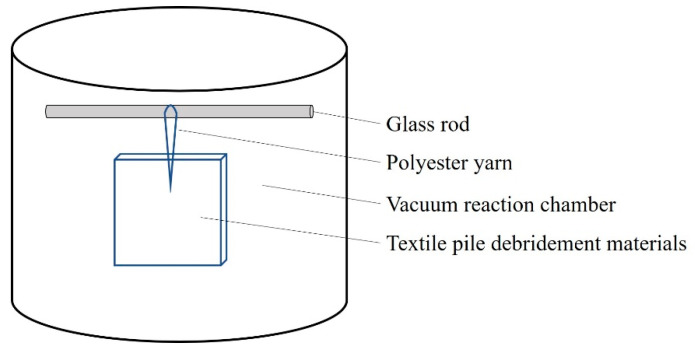
Illustration of the plasma treatment setup.

A series of sliver knitted pile fabrics with a double faced structure were manufactured on a circular knitting machine, followed by back-coating, thermosetting, and shearing, as reported in our previous work [13,17]. In order to further improve the liquid absorption properties of the textile pile debridement material, low temperature plasma surface modification was applied with a plasma treatment instrument (HD-300, Zhongkechangtai Plasma Technology Co., Ltd., Changzhou, Jiangsu, China). The textile pile debridement material was first cut into the size of 10 cm × 10 cm, followed by 30 min of ultrasonic cleaning in acetone and rinsing in distilled water three times. After becoming completely dry in a vacuum drying oven (DZF-6030B, Shanghai Yiheng Technology Co., Ltd.), samples were put into the vacuum reaction chamber (height 30 cm and diameter 30 cm) of the plasma treatment instrument under the power of 150 W for 1, 2, 3, 4, and 5 min in atmospheres of oxygen or helium. The plasma treatment setup is illustrated in Figure 1.

### 2.3. Surface Micromorphology Analysis

Scanning electron microscopy (SEM) and atomic force microscopy (AFM) were used to inspect changes in the surface morphology of samples before and after plasma treatment. The micrographs were obtained from TM-3000 SEM (Hitachi, Japan) with an accelerating voltage of 15 kV and a FastScan AFM (Bruker Nano Surface, USA) with a Scanasyst-Air probe in light tapping, scanning, and imaging mode. The contact force was 0.4 cN/m and the resonance frequency was 70 kHz. Finally, the surface topography of samples with an area of 4 μm × 4 μm were obtained. The root mean square roughness (Rq), average roughness (Ra), and the maximum peak valley roughness (Rmax) were used as indexes to analyze the microstructure of the sample surface.

### 2.4. Surface Chemistry Analysis

An X-ray photoelectron spectrometer (XSAM800, Kratos Amicus, Manchester, UK) was used to investigate the surface chemical composition and chemical groups of the textile pile debridement material. An Al-Kα (hv = 1486.6 eV) X-ray was selected as the excitation source. Peaks of the C 1s spectrum were fitted using XPS peak 4.1 software to analyze the percentage content of the chemical functional groups on the surface of each sample.

### 2.5. Water Uptake Capacity

A water absorption study was conducted to estimate the exudate holding capacity of the textile pile debridement material before and after plasma treatment, according to ISO 9073-6. Three dry samples with a size of 1 cm × 1 cm were immersed in distilled water at room temperature for 10 s, then were taken out and drained for 10 s to remove excess water. The water uptake capacity (*C_w_*) was quantified by the following equation, where *w*_0_ and *w*_1_ are the dry and wet weight of the samples, respectively.

(1) $$C_{w}=\frac{w_{1}-w_{0}}{w_{0}}\times 100$$

### 2.6. Mechanical Characterizations

**Figure 2:**
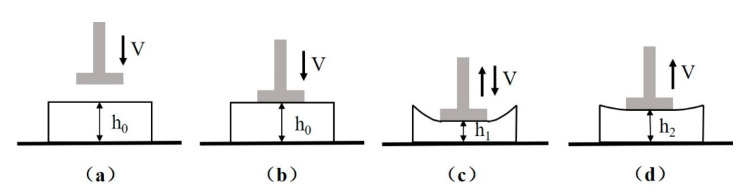
Diagram of the compression test process: (**a**) initial state, (**b**) presser contact with sample, (**c**) presser drop at lowest position, (**d**) final state.

The compression strength and elastic recovery rate were employed to evaluate the compression properties of the textile pile debridement material in both dry and wet states. The typical test process of a LLY-06D compression tester (Laizhou Electron instrument Co., Ltd., Laizhou, China) is exhibited in Figure 2, where *h*_0_ is the original thickness of the sample and *h*_1_ equals half of *h*_0_. The diameter of the presser (d) was designated as 1 cm, and the velocity of the presser (v) was set as 1 cm/min. Compression strength was obtained when the presser dropped to its lowest position, as illustrated in Figure 2c. Elastic recovery rate (E) refers to the percentage of elastic deformation in the total deformation [13,18], which can be calculated according to formula (2).

(2) $$E\;\left(\%\right)=\frac{h_{2}-h_{1}}{h_{0}-h_{1}}\times 100$$

### 2.7. Coagulation Assay

The coagulation activity of the textile pile debridement material was evaluated using a kinetic clotting time method. Commercial gauze and glass cover slips were taken as commercial and positive controls, respectively. Specimens measuring 10 mm × 10 mm were placed in 24-well tissue culture plates in triplicate. Afterwards, 10 μL mixed solution of whole human blood and trisodium citrate with a volume ratio of 9:1 were dropped onto the surface of each specimen, followed by a 5 μL CaCl_2_ solution of 0.2 mol/L to initiate the blood coagulation cascade. After incubation at 37 °C for a predetermined period of time, specimens were inundated with 2.5 mL distilled water at the end of 5, 10, 30, and 60 min, and placed back into the chamber for another 5 min. Then 200 μL resultant blood solution were transferred into a 96-well plate, and the optical density at 540 nm was obtained on an ELISA plate reader (Multiskan FC, Thermo). To prepare samples for SEM observation, specimens were fixed in 2% glutaraldehyde solution at 4 °C for 30 min, dehydrated with a series of gradient alcohol solution of 55%, 70%, 80%, 90%, 95%, and 100%, and then dried at 4 °C.

### 2.8. In Vitro Cytotoxicity

The in vitro cytotoxicity of the textile pile debridement material was evaluated by an MTT assay [19]. Fluid extracts were prepared from 0.2 g of textile pile debridement material and medical gauze in 2 mL DMEM at 37 °C for 24 h. L929 fibroblasts were seeded into a 96-well plate at a density of 5 × 10^3^ per well and cultured for 4 h. Then the culture medium was replaced by fluid extracts and cultured for 1, 3, 5, and 7 days. Twenty microliters of MTT solution were added to each well and incubated for 4 h. After discarding the supernatant, 100 μL of DMSO were added to each well before measuring the optical density (OD) at 570 nm with a microplate reader (Multiskan FC, Thermo, Shanghai, China).

### 2.9. In Vitro Biofilm Removal Test

#### 2.9.1. Formation of *S. aureus* Biofilm

An isolated single bacterial colony picked from an agar plate was transferred to 4 mL of TSB medium and then incubated at 37 °C for 14 h at a speed of 180 rpm. An overnight culture of *S. aureus* was diluted in TSB to 10^6^ cfu/mL. Then 600 μL diluted bacterial liquid were added into each well of 24-well plates with cell growing glass slides (Zhida Experiment Equipment, Nantong, China). Cells were cultured at 37 °C for 24 h. At the end of incubation, the supernatant was removed and the formed biofilms were washed with phosphate buffered solution (PBS, pH = 7.2) to remove planktonic and loosely attached bacteria.

#### 2.9.2. Setup of Biofilm Removal Test

**Figure 3:**
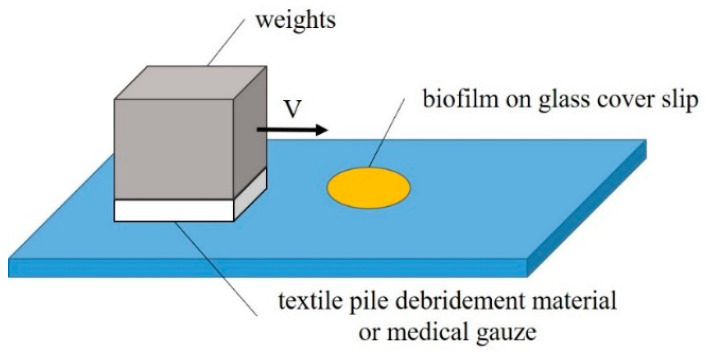
Experiment setup of the in vitro removal of biofilm.

An in vitro biofilm removal test was conducted on a self-built experimental device under different velocities and pressures in both dry and wet states, as illustrated in Figure 3. The whole experiment was completed in a sterile environment. The wiping speed was selected from two levels: fast (2 cm/s) and slow (1 cm/s). The pressure, adjusted by the loading weight and sample area, was set as high (15.68 cN/cm^2^), medium (12.25 cN/cm^2^), or low (8.71 cN/cm^2^). PBS solution was used to fully soak the debridement material or cotton gauze in the wet state test.

#### 2.9.3. Evaluations of Biofilm Removal

An MTT cell viability assay, scanning electron microscopy (SEM), and confocal laser scanning microscopy (CLSM) were employed to compare the viability and morphology before and after the biofilm removal test.

- MTT cell viability assay

An MTT cell assay was used to assess the viability of bacteria in the biofilm. Only live bacteria in the biofilm were counted in the MTT assay, by measuring the metabolic activity of each individual bacterial cell [20]. Cell growing glass slides, before and after the biofilm removal tests, were put back into 24-well plates and incubated in 0.05% MTT at 37 °C for 2 h. After washing, the purple formazan that formed inside the bacterial cells was dissolved by isopropanol and then measured by a microplate reader (Multiskan FC, Thermo, Shanghai, China) at 570 nm. Three parallel samples were set up in each group.

- SEM of *S. aureus* biofilm

For the visualization of the *S. aureus* biofilm architecture, SEM images were taken. After fixation by glutaraldehyde for 2 h at 4 °C, samples, before and after the biofilm removal tests, were dehydrated by a series of ethanol with increasing concentrations (30%, 50%, 70%, 80%, 90%, 95%, and 100%) for 10 min each time. Dehydrated specimens were coated with gold–palladium and observed.

- CLSM of *S. aureus* biofilm

Biofilms were prepared and fixed by glutaraldehyde for 2 h at 4 °C. After washing three times with PBS, cells within the biofilm were fluorescently stained in the dark at 4 °C by 50 μg/mL FITC for 30 min and 10 μg/mL PI for 15 min, which identified polysaccharide and DNA in the biofilm structure, respectively [21]. Biofilm morphology was imaged using CLSM.

## 3. Results and Discussion

### 3.1. SEM Observation

**Figure 4:**
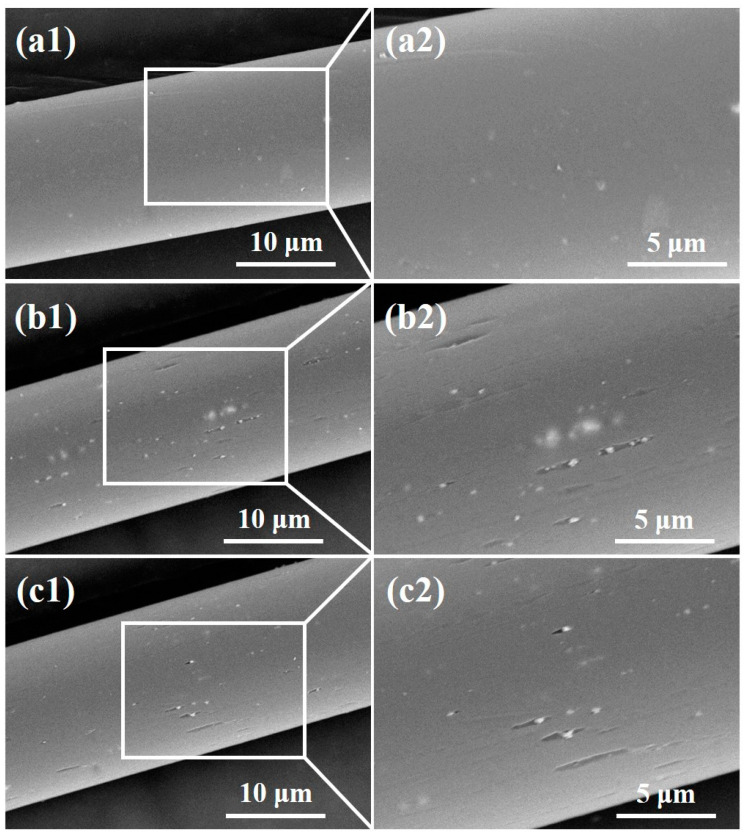
SEM images of the polyester fiber surface (**a**) before and after (**b**) oxygen, (**c**) helium plasma treatments.

Figure 4 shows SEM images of the polyester fiber surface before and after plasma treatment. It can be clearly seen that the surface of the pile fiber without plasma treatment is relatively smooth, but after oxygen or helium plasma treatment, the surface of the pile fiber becomes much coarser. Grooves parallel to the fiber axis and granules were found on the surface, indicating that the oxygen and helium plasma treatment damaged the surface morphology of the pile fibers. This may be caused by the physical etching of plasma modification [22]. A large number of active particles were generated during plasma discharge, which bombarded the fiber surface, leading to the gasification of some solids on the fiber and the formation of micro grooves, as shown in Figure 4. It was also reported that plasma treatment destroys the chemical chain segment of polymers, causing a degradation reaction of macromolecules and the generation of new small molecular substances. These small molecules remained on the surface or were wrapped in the active particles, forming white particles, as shown in Figure 4. In addition, the impact of active particles during plasma treatment destroyed the crystalline area in the polyester macromolecular structure, changing it into the disordered amorphous region, resulting in a relatively obvious morphological change. The comparative analysis of oxygen and helium plasma treatments showed that the number of grooves and granules on the fiber surface in Figure 4b is relatively higher than that in Figure 4c, indicating that oxygen plasma has a relatively higher effect than helium plasma does. This is due to the fact that oxygen is chemically active, while helium is a kind of inert gas. The difference in chemical properties leads to distinctions in the surface modification mechanism and energy during the plasma treatment.

### 3.2. AFM Observation

**Figure 5:**
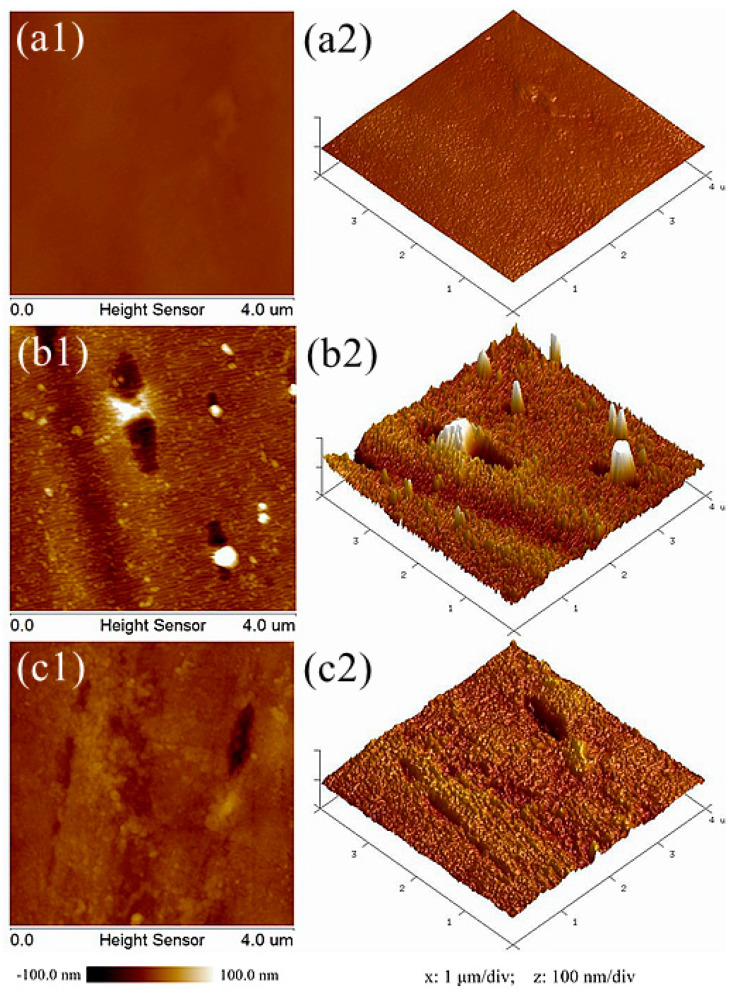
AFM images of polyester fiber surface (**a**) before and after (**b**) oxygen and (**c**) helium plasma treatments.

Figure 5 shows AFM images of the polyester fiber surface before and after plasma treatment. Comparing the two-dimensional and three-dimensional AFM morphologies of the three samples, it can be seen that the surface of the sample without plasma treatment is relatively smooth and flat. However, after oxygen plasma treatment, obvious granular substances (white highlights in Figure 5b1), conical bulges (Figure 5b2), strip grooves (black strips in Figure 5b1), and concave parts (Figure 5b2) were observed on the fiber surface. For helium plasma treatment, similar morphological changes, to a lesser degree, were found. Furthermore, NanoScope Analysis software was selected to quantitatively analyze the surface roughness. The root mean square roughness (Rq), average roughness (Ra), and the maximum peak valley roughness (Rmax) of different samples are listed in Table 1. The Rq value of the samples without plasma treatment is 4.19 nm, while the Rq value of the samples after oxygen or helium plasma treatment are 18.8 nm and 8.41 nm, respectively. The growth rates are 348.69% for oxygen and 100.72% for helium plasma treatment. The same upward trend also occurred in the parameters of Ra and Rmax. All the results revealed that the surface roughness of fibers increased significantly after plasma treatment. Besides, the impact of oxygen plasma is more obvious than that of helium plasma. The results of AFM are consistent with that of SEM. The high-energy particles bombarded or collided on the fiber surface during plasma treatment, transferring energy into the macromolecules on the fiber surface, which broke the primary chemical bonds, degraded the chain segments, and generated volatile gases [23]. In addition, active oxygen atoms and free radicals in the plasma can also introduce new cross-links to induce oxidation reactions on the fiber surface [24]. These effects not only change the micromorphology of the fiber surface, but also improve the roughness greatly. Meanwhile, it has been reported that the improvement of fiber surface roughness helps to enhance the hydrophilicity of the fiber [25,26], which gives a better explanation for why the water absorption property of the textile pile debridement material after plasma treatment was significantly improved.

### 3.3. XPS Analysis

**Figure 6:**
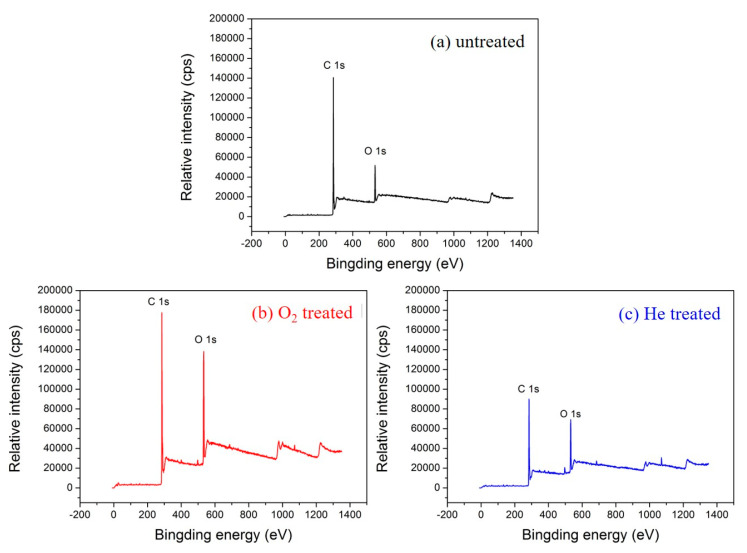
XPS overall scans of the (**a**) untreated, (**b**) oxygen, and (**c**) helium plasma-treated textile pile materials.

XPS overall scans of the untreated and plasma-treated textile pile materials are illustrated in Figure 6. Compared with the control sample without plasma treatment, the intensity of the O 1s peak increased significantly after the treatment with oxygen or helium plasma. Table 2 exhibits the relative chemical composition and atomic ratios before and after plasma treatment. The O/C atomic ratio before plasma treatment was 0.17, while that of the sample after oxygen and helium plasma treatment reached up to 0.32 and 0.27, respectively, which are 90.94% and 63.42% higher than that of the control sample. The results demonstrate that oxygen-containing polar groups were introduced into the fiber surface via plasma treatment. Moreover, the amount of oxygen-containing groups introduced by oxygen plasma is higher than that of helium plasma.

**Figure 7:**
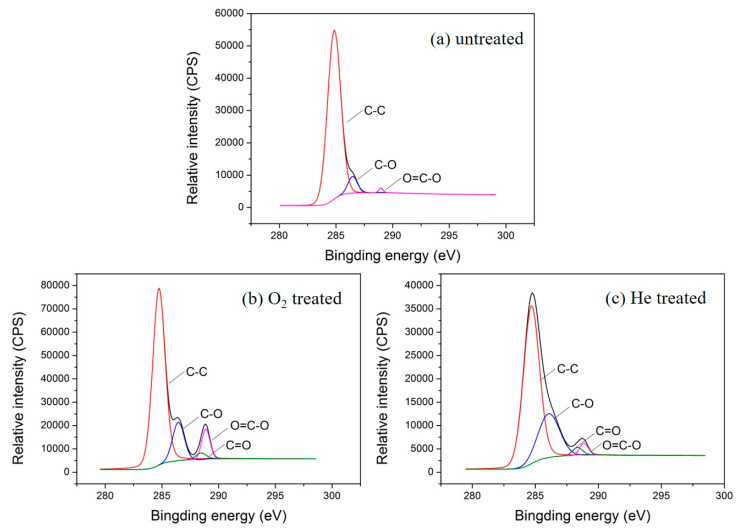
C 1s spectra of the (**a**) untreated, (**b**) oxygen, and (**c**) helium plasma-treated textile pile materials.

To further explore the types of polar groups introduced on the fiber surface by plasma treatment, XPS peak 4.1 software was employed to analyze the C 1s photoelectron scanning pattern. The C 1s spectra and functional group percentage of the untreated and plasma treated textile pile materials are demonstrated in Figure 7 and Table 3. It can be seen from Figure 7 that the C 1s spectrum of the sample without plasma treatment consists of three peaks, i.e., C-C, C-H groups at 284.6 eV, C-O, C-OH groups at 286.1 eV, and O=C-O, COOH groups at 288.9 eV [27]. However, after oxygen or helium plasma treatment, the C 1s spectrum shows a new peak at 288.5 eV, which corresponds to the functional group of C=O. In addition, the content percentage of carbon groups on the fiber surface declined obviously after plasma treatments. The content of C-C and C-H groups on the fiber surface of the control sample was as high as 92.90%, while it dropped to 75.45% and 69.57%, respectively, after the oxygen or helium plasma treatments. Meanwhile, the percentage of oxygen-containing groups (C-O, C-OH, C=O, O=C-O, COOH) rose distinctly after plasma treatment. The percentage of O=C-O and COOH grew to 8.00% after the oxygen plasma treatment, which was more than three times higher than that of the helium plasma treatment (2.47%). This demonstrates that the C-C chemical bonds broke and reacted with the oxygen atoms under the action of plasma, resulting in the formation of new oxygen-containing polar groups [28]. The oxidation of the active substances during plasma treatment increases the number of hydrophilic groups, which is beneficial for the enhancement of the liquid absorption property of the textile pile debridement material.

### 3.4. Water Uptake Capacity

**Figure 8:**
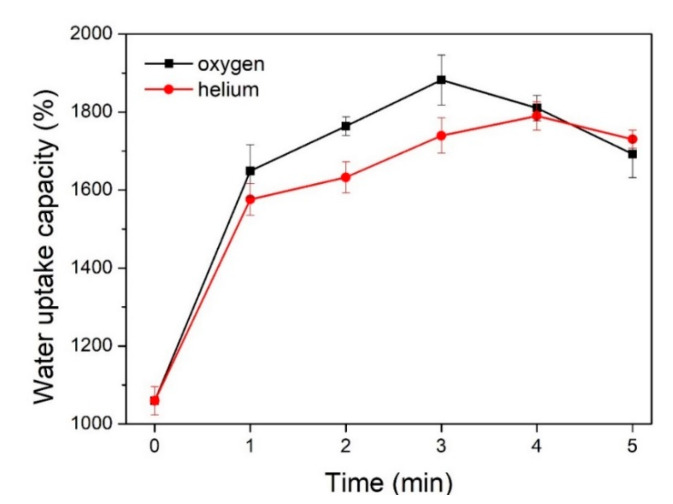
Water uptake capacity before and after plasma treatments.

The influence of the plasma treatment parameters on the water uptake capacity of the textile pile debridement material is shown in Figure 8. It can be observed that the water uptake capacity of the samples after the plasma treatments is apparently higher than that of the control sample without treatment, verifying the positive impacts of plasma treatment on the water uptake capacity of the textile pile debridement material. In addition, oxygen plasma has a more sensitive effect than helium plasma, which could be attributed to the introduction of oxygen-containing polar groups and the enhancement of the surface energy of the polyester fiber [29]. Meanwhile, the water uptake capacity of the samples treated by oxygen and helium plasma first rose and then fell with the extension of the treatment time, and peak values were achieved at 3 min for oxygen and 4 min for helium plasma. Therefore, the optimum parameters of plasma treatment are oxygen atmosphere, at a power of 150 W, for 3 min. All the subsequent measurements, including mechanical properties, coagulation activity, and the in vitro biofilm removal test, are performed on samples after plasma treatment with the above optimum parameters.

### 3.5. Compression Strength and Elastic Recovery Rate

**Figure 9:**
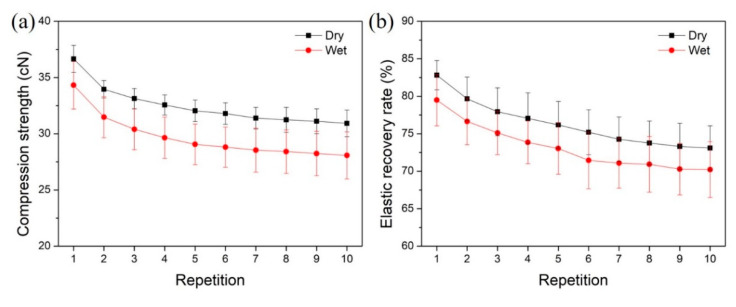
Influence of repetition on (**a**) compression strength and (**b**) elastic recovery rate of textile pile debridement materials in dry and wet states.

To achieve effective removal of necrotic tissues, it is always necessary to wipe the wound with textile pile debridement material several times. Therefore, a repeated compression test was performed ten consecutive times to study the impact of repetition on the compression strength and elastic recovery rate of the textile pile debridement material. The compression strength and elastic recovery rate of the textile pile debridement material in dry and wet states at each compression time are displayed in Figure 9. It is apparent that the wet compression strength is relatively lower than the corresponding dry compression strength. The same trend can be found in the results of the elastic recovery rate. This may be caused by the swelling effect of the fibers and the interaction of water molecules among each pile fiber, which make the fibers more easy to compress and makes recovery harder. Moreover, both the compression strength and elastic recovery rate declined slightly with repetition. This is due to the fatigue properties of textile fibers, i.e., mechanical strength reduced with the repetition of action. However, with the increasing compression repetition, the descent shrinks and the curves tend to be stable.

To further quantificationally study the influence of the wet condition and repetition on the compression strength and elastic recovery rate of the textile pile debridement material, the percentage of wet/dry compression strength and elastic recovery rate under different compression repetitions are listed in Table 4. The percentages of wet/dry compression strength at different repetitions all exceed 90%, while the percentages of wet/dry compression elastic recovery rate is maintained above 95%. In other words, the wet compression strength and elastic recovery rate are only 5–10% lower than that in a dry state, which demonstrates that the wet condition seldomly affects the practical application of the textile pile debridement material. Table 5 highlights the retention rates of compression strength and elastic recovery rate in dry and wet states under different compression repetitions. The retention rates of both compression strength and elastic recovery rate show an evident descending trend as the number of repetitions increases. However, after 10 compressions, the strength and elastic recovery rate are still higher than 80% of the initial data, indicating that the textile pile debridement material can provide enough strength and elasticity during practical application even after 10 wipes.

### 3.6. Coagulation Activity

**Figure 10:**
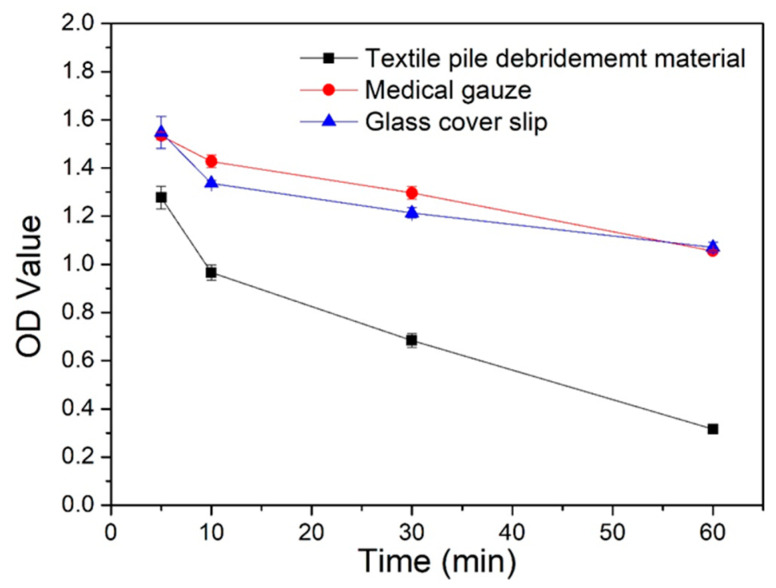
OD values of the coagulation assay for different samples.

The absorbance OD value at 540 nm of aqueous blood solutions at different time points is displayed in Figure 10. The lower the absorbance value, the less the content of free hemoglobin in the blood aqueous solution, signifying a better blood clotting efficiency. The absorbance values of textile pile debridement material at all time points were significantly lower than that of medical gauze and glass cover slips, which implies that the textile pile debridement material has a faster coagulation rate compared to gauze and glass cover slips. The OD value of the debridement material at 5 min is similar to the value of the glass cover slip at 30 min. In other words, the blood clotting effect of the glass cover slip at 30 min will be achieved by the textile pile debridement material within 5 min, suggesting that the textile pile debridement material has a higher ability to promote blood coagulation compared to the glass cover slip, which is always considered as a material with a high coagulation performance [30]. This may have resulted from the distinct three-dimensional structure of debridement materials. Pile fibers in contact with blood are densely and vertically arranged, forming irregular channels, which increase the contact space between the blood and fibers. Meanwhile, the structure of gauze is two-dimensional. In addition, the gaps among the fibers are relatively large and independent, which is not beneficial for blood clotting.

**Figure 11:**
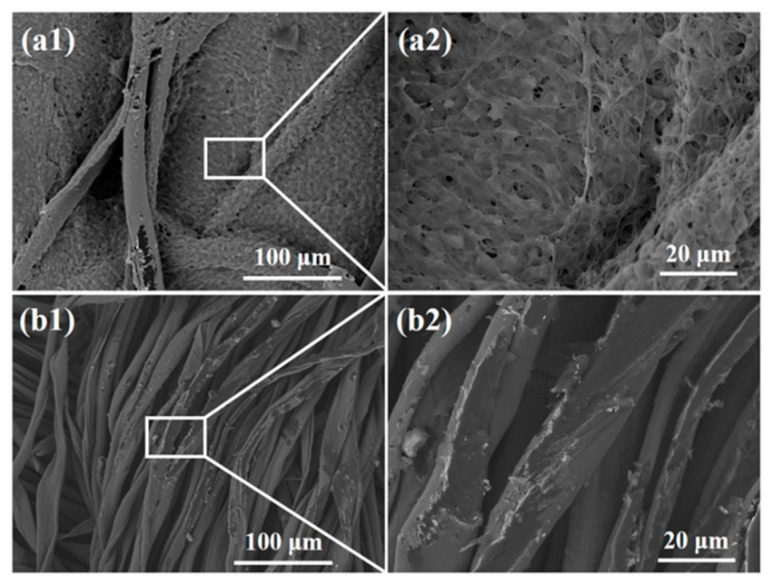
SEM images of (**a**) the textile pile debridement material and (**b**) medical gauze at 30 min in the blood coagulation test.

SEM images of the textile pile debridement material and gauze at 30 min in the blood coagulation test are shown in Figure 11. A dense fibrin net containing a large number of blood cells was observed among the fibers of the textile pile debridement material. Meanwhile, fibers attached to scarce red blood cells among the threads of the gauze. The SEM results confirm that the blood clotting reaction was completed on the textile pile debridement material. The fibrinogen in the plasma was transformed into a fibrin monomer, which formed stable long-chain fibrin polymers. The interlacing of the fibrin polymers constituted a complex cross-linking network, which macroscopically appeared as clots.

### 3.7. In Vitro Cytotoxicity

**Figure 12:**
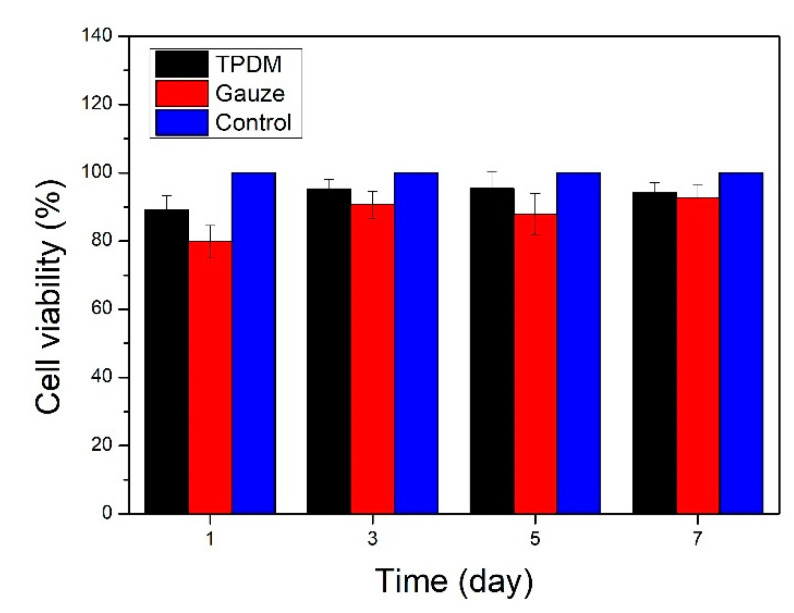
In vitro cytotoxicity results of the textile pile debridement material and medical gauze.

The relative cell viability was employed to evaluate the in vitro cytotoxicity results of the textile pile debridement material. Cell viability was expressed as the percentage of the OD value of the experimental group to the control group. Figure 12 shows the in vitro cytotoxicity results of the textile pile debridement material and medical gauze. It can be seen that the cell viability of the textile pile debridement material at different times are all above 85% and are higher than for medical gauze, indicating that the textile pile debridement material has no obvious cytotoxic effect. However, in the future, a direct cultivation of the cells onto the textile pile debridement material’s surface is necessary to evaluate the pile material’s scratching effect on cells and to avoid any damage to the healing tissue.

### 3.8. Results of Biofilm Removal Test

#### 3.8.1. MTT Cell Viability Assay

**Figure 13:**
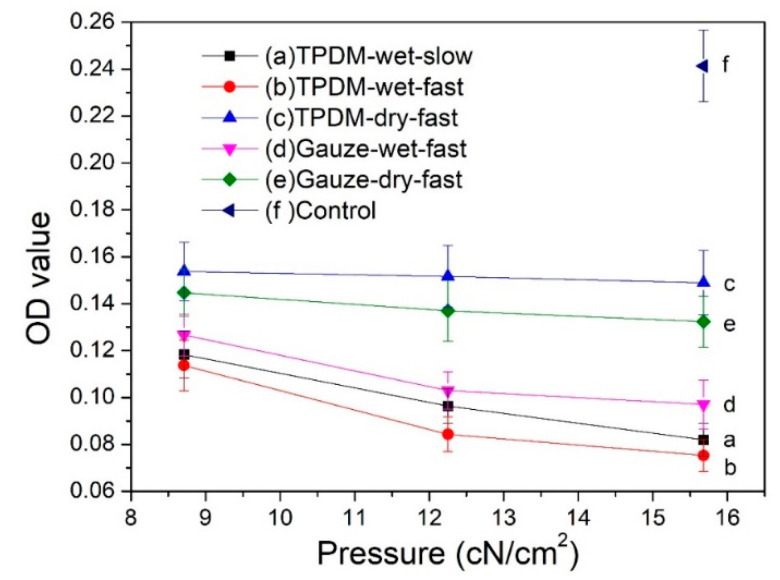
MTT results for in vitro biofilm removal tests.

The MTT results for the in vitro biofilm removal test are shown in Figure 13. Only viable bacteria are counted by measuring the metabolic activity of individual bacterial cells in the MTT method. Therefore, a high absorbance OD value suggests a strong bacterial activity and a high number of living bacteria in the biofilm. It has been reported that an OD value exceeding 0.24 represents a strain with strong biofilm productivity; a value between 0.12 and 0.24 refers to a poor producer; an absorbance below 0.12 indicates a strain without the ability to yield biofilm [31].

It can be seen from Figure 13 that the OD value of samples before the biofilm removal test was higher than 0.24, implying a biofilm with vigorous viability. All five curves after the removal tests have a significantly lower value than that of the control, suggesting an evident reduction in biofilm. By analyzing the influence of applied pressure on the biofilm removal, it can be seen that all five curves in Figure 13 show a descending trend, which indicates that the increase in applied pressure is beneficial for removing biofilm. The OD values obtained in the dry condition (curves c and e) are obviously higher than that obtained in the wet condition (curves d, a, and b), which specifies that the wet condition is preferred for the removal of biofilm. Moreover, the OD values of the textile pile debridement material in a wet state (curves a and b) are lower than that of medical gauze (curve d), illustrating that the textile pile debridement material has a much better biofilm removal performance than that of medical gauze. Furthermore, when exploring the impact of wiping speed, the absorbance values for a slow wiping speed (curve a) is found to be slightly higher than that for fast wiping (curve b) under the same pressure, indicating that a high speed is recommended in the practice of wound debridement. Besides, the absorbance OD values on curves a and b are all lower than 0.12, regardless of the pressure and velocity. In other words, when the textile pile debridement material is used for biofilm removal in a wet state, whether the wiping velocity is fast or slow, the original structure of the biofilm can be destroyed and its biological activity can be significantly reduced.

#### 3.8.2. SEM of *S. aureus* Biofilm

**Figure 14:**
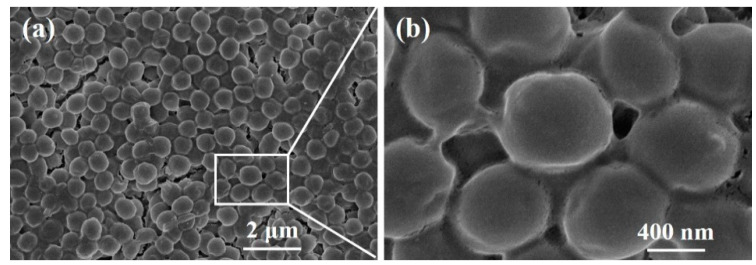
SEM images of a biofilm before debridement: (**a**) 10000×, (**b**) 50000×.

**Figure 15:**
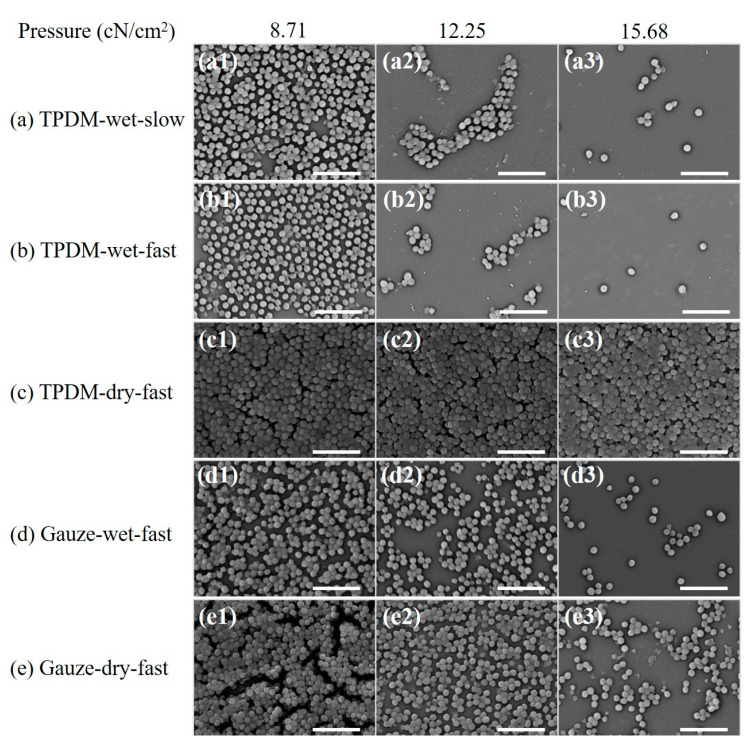
SEM images after *in vitro* biofilm removal tests (scale bar: 5 μm): (**a**)TPDM-wet-slow, (**b**) TPDM-wet-fast, (**c**) TPDM-dry-fast, (**d**) Gauze-wet-fast, (**e**) Gauze-dry-fast.

The morphologies of biofilms before and after debridement are shown in Figure 14 and Figure 15, respectively. The biofilm in Figure 14a is made up of spherical bacteria closely arranged to each other, forming a multi-layered stacked structure. Meanwhile, a slimy barrier and adhesion are clearly observed among bacterial colonies under a high magnification view in Figure 14b. The extracellular polysaccharide-wrapped bacterial colonies exhibit the typical features of biofilms [32], certifying a successful construction of an in vitro biofilm model.

The SEM morphology results of biofilms under different in vitro debridement conditions (Figure 15) are consistent with the MTT results. Compared with Figure 14, the bacterial densities of all the SEM images in Figure 15 show a decrease to a certain extent, which manifests that physical debridement is an effective method to remove biofilms. It can be clearly seen that the number of bacterial colonies in Figure 15 goes down in various levels with the increase in pressure, except for Figure 15c. This demonstrates that pressure is positive for the removal of biofilms. Compare the results between dry and wet conditions. The bacterial population in dry debridement (Figure 15c,e) is obviously larger than that in wet conditions (Figure 15a,b,d), especially for the textile pile debridement material (Figure 15c). Meanwhile, for medical gauze, although there are still stacked bacterial colonies, noticeable cracks and separations can be observed under a pressure of 8.71 cN/cm^2^ (Figure 15e1). Moreover, with the increase in applied pressure, a single layer of spherical bacteria is found and the bacterial distribution density gradually decreases in Figure 15e2,e3. This may be caused by diversity in the structure and thickness of the textile pile debridement material and medical gauze. The textile pile debridement material designed in this study is a kind of three-dimensional pile fabric with a high thickness (about 10 mm), while medical gauze is eight layers of woven fabric in a plain structure with a much lower thickness (approximately 3 mm). In addition, the pile fibers in the textile debridement material are vertical in direction, i.e., parallel to the compression action, leading to an absorption and cushioning effect, as well as a poor pressure force delivery from top to bottom. Therefore, the effect of the textile pile debridement material during dry debridement is inferior to that of medical gauze, and the amount of bacterial colonies does not change significantly under different pressures. For the SEM results under wet conditions, the number of residue bacterial colonies in Figure 15b is obviously less than that in Figure 15d, indicating that the textile pile debridement material has a much better biofilm removal effect than medical gauze. Further exploring the impact of wiping speed, it can be seen that the residual bacteria population after fast debridement (Figure 15b) is noticeably lower and more scattered than those debrided at a slow speed (Figure 15a). Thus, a high debridement speed is recommended for the practice of biofilm removal.

#### 3.8.3. CLSM of *S. aureus* Biofilm

**Figure 16:**
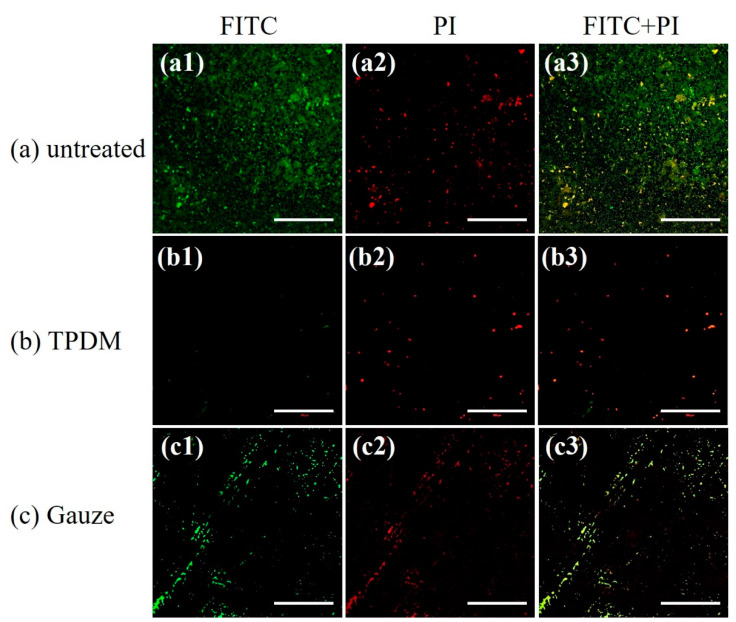
CLSM images for in vitro biofilm removal tests (scale bar: 200 μm).

CLSM images of biofilms debrided with the textile pile material and gauze under a pressure of 15.68 cN/cm^2^, as well as the control before debridement, are illustrated in Figure 16.

FITC is a green nucleic acid probe with the ability to make polysaccharide in an extracellular matrix green. PI is a red fluorescent dye which can only stain DNA in bacteria. It can be clearly observed from Figure 16a that the field of vision is full of *S. aureus* stained in green, suggesting the typical characteristic of glycocalyx formation in the biofilm. In contrast, green bacteria are barely observed in Figure 16b, indicating a satisfactory removal of biofilm through the action of textile pile debridement material. For biofilms treated with medical gauze, scratches are obviously found and the green area is much smaller than that of the control biofilm, which indicates that the debridement effect of medical gauze is not as good as that of the textile pile debridement material. All the CLSM results are consistent with the results acquired by MTT and SEM.

In addition, it was reported that a silver-containing dressing is the first choice to treat chronic wound biofilms, which can remove 90% of the bacteria within 24 h and eradicate biofilms within 48 h [33]. Besides, the effect of a novel gelling fiber dressing with silver was examined on a porcine wound biofilm model. The results show that a gelling fiber dressing with silver exhibited the significant reduction and detachment of Pseudomonas aeruginosa biofilms from wounds after 3 days [34]. The results of the in vitro biofilm removal test show that most of the biofilm can be removed by the repeated wiping actions of the textile pile debridement material on the wound, which is considered to be a more convenient approach to treat wound infections.

## 4. Conclusions

Herein, we prepared a novel textile pile debridement material treated by plasma. The experimental results indicated that the hydrophilicity and water uptake capacity of the textile pile debridement material were improved by plasma treatment. Meanwhile, the textile pile debridement material displayed satisfactory mechanical properties in wet conditions. Moreover, the textile pile debridement material also presented an ideal blood clotting performance and excellent biofilm removal capacity. The novel textile pile debridement material will find its potential application in wound debridement.

## Figures and Tables

**Table 1:** Roughness of the fiber surface before and after plasma treatments.

Samples	Root Mean Square Roughness Rq (nm)	Average Roughness Ra (nm)	Maximum Peak Valley Roughness Rmax (nm)
(a) Before plasma treatment	4.19	3.02	46.7
(b) O_2_ plasma treatment	18.8	11.3	282.0
(c) He plasma treatment	8.41	6.08	97.5

**Table 2:** Relative chemical composition and atomic ratios of textile pile materials before and after plasma treatment.

Samples	Chemical Composition		Atomic Ratio of O/C
	C 1s (%)	O 1s (%)	
(a) Before plasma treatment	84.91	14.08	0.17
(b) O_2_ plasma treatment	75.01	23.75	0.32
(c) He plasma treatment	77.42	20.98	0.27

**Table 3:** Functional group percentage before and after plasma treatments.

Electron Binding Energy (ev)	Functional Groups	(a) Before Plasma Treatment (%)	(b) O_2_ Plasma Treatment (%)	(c) He Plasma Treatment (%)
284.6	C-C, C-H	92.90	75.45	69.57
286.1	C-O, C-OH	6.33	14.36	25.75
288.5	C=O	0	2.19	2.21
288.9	O=C-O, COOH	0.77	8.00	2.47

**Table 4:** Percentage of wet/dry compression strength and elastic recovery rate under different compression repetitions.

Compression Repetition	Percentage of Wet/Dry Compression Strength (%)	Percentage of Wet/Dry Elastic Recovery Rate (%)
1	93.60	95.98
2	92.69	96.22
3	91.76	96.39
4	91.05	95.85
5	90.69	95.88
6	90.61	95.02
7	90.92	95.71
8	90.94	96.15
9	90.77	95.87
10	90.78	96.06

**Table 5:** Retention rate of compression strength and elastic recovery rate in dry or wet states under different compression repetitions.

Compression Repetition	Retention Rate of Compression Strength (%)	Retention Rate of Elastic Recovery Rate (%)
1	100.00	100.00
2	92.62	91.72
3	90.36	88.58
4	88.81	86.39
5	87.40	84.68
6	86.72	83.95
7	85.62	83.17
8	85.21	82.79
9	84.86	82.29
10	84.35	81.80
